# Forgone Health and Economic Benefits Associated with Socioeconomic Differences in Organized Cervical Cancer Screening

**DOI:** 10.31557/APJCP.2019.20.12.3755

**Published:** 2019

**Authors:** Vivian Chia-Rong Hsieh, Zih-Jhen Chen, Chin-Chen Liu, Jen-Huai Chiang, Shwn-Huey Shieh, Ming-Shun Hsieh

**Affiliations:** 1 *Department of Health Services Administration, China Medical University, *; 3 *Department of Nursing, China Medical University Hospital, Taichung, *; 2 *Department of Family Medicine, E-Da Hospital, Kaohsiung, *; 4 *Institute of Occupational Medicine and Industrial Hygiene, College of Public Health, National Taiwan University, *; 6 *Department of Emergency Medicine, Taipei Veterans General Hospital, *; 7 *School of Medicine, National Yang-Ming University, Taipei, *; 5 *Department of Emergency Medicine, Taipei Veterans General Hospital, Taoyuan Branch, Taoyuan, Taiwan. *

**Keywords:** Pap smear, cancer screening, cervical cancer, inequalities, health, economics

## Abstract

**Objective:**

To describe cervical cancer screening participation among women in Taiwan under its population-based screening policy and to estimate the economic burden of disease attributable to avoidable disparities in cervical cancer (CC) screening.

**Methods:**

We identified a nationally-representative sample of females aged 30 years or above who were eligible for Pap smear testing in Taiwan from 1 January to 31 December 2013. An administrative database with detailed claims of health care utilization under the universal coverage health care system was used. Socioeconomic position of the female subjects was defined using the occupation classification, and two groups were specifically identified: general (O1) and low-income (O5) groups. Differences in screening rate, CC prevalence, and CC-attributable deaths were assessed between the two groups. Economic consequences as a result of screening inequalities were estimated using actual total health care spending (health care expenditure), monetary value per life-year and years of life lost for ill health and screening disparities (health as consumption good), and productivity losses alongside costs of social benefits (health as capital good).

**Result:**

A total of 301,057 enrolled females aged 30 years and older eligible for screening were identified. Overall, 3-year and 1-year screening rates among all subjects were 0.601 and 0.372, respectively. Impact of observed differences in screening translated to US$59,568 of health care spending in one year, 90.4% of which was specific to hospital admissions. When we viewed health as a consumption good and capital good, the impact of screening disparity on health losses through CC would be equivalent to US$78,095 and US$190,868, respectively.

**Conclusion:**

Forgone health and economic benefits associated with inequalities in CC screening uptake can be considerable in productive women.

## Introduction

A plethora of literature has highlighted the long-term benefits of cervical cancer (CC) screening in females (Andrae et al., 2008; Chiang et al., 2010; Ko et al., 2015; Felix et al., 2016). Since CC can be readily detected before becoming lethal at early stage with screening, adequate uptake of such preventive interventions can greatly reduce the health and economic burden of CC. The sensitivity of CC screening tests in countries like Taiwan can be as high as 82%, and once CC is detected early, there is a 92% of survival following successful treatment and prevention of adverse health outcomes (Chiang et al., 2016; Chao et al., 2008). Unfortunately, improvement in screening rate is still much needed for many countries (Akinyemiju, 2012). 

CC screening methods currently include the Papanicolaou smear (Pap test) and high-risk human papillomavirus (HPV) testing. Screening strategies and their coverage, however, vary with context. In Taiwan, population-based organized screening with Pap test is subsidized every three years for women 30 years and over. By definition, organized screening is characterized by its universal coverage for eligible individuals who are invited to participate after their risks have been objectively assessed (Walsh et al., 2011). Contrary to other countries like England and the United States (Goodwin et al., 2011; Kepka et al., 2014; Massat et al., 2015), Taiwan observes only a modest coverage of cancer screening services despite government subsidization (51.0% in 2007 and 55% in 2010) (Chen et al., 2009; Chiou and Lu 2014). Nevertheless, age-standardized CC incidence rate was reported to be declining, from 24.24 per 100,000 in 2002 to 12.99 per 100,000 in 2012; five-year age-standardized survival rate was 74.9% (Chiang et al., 2016). These rates are comparable with other countries like the United States (age-standardized incidence: 7.6 per 100,000; 5-year survival rate: 67%) (American Cancer Society, 2019), United Kingdom (age-standardized incidence: 10 per 100,000; 5-year survival rate: 67%) (Cancer Research UK, 2019) and the Netherlands (age-standardized incidence: 8.2 per 100,000; 5-year survival rate: 71%) (Bulk et al., 2003). It was believed that screening coverage is partly hindered by barriers such as stigma for seeing gynecologists and conducting ‘invasive’ examinations, little access to female providers (Lofters et al., 2011), never been advised by doctors the benefits of screening, and lack of intention from doctors to examine unless patient is sick (Todorova et al., 2009). 

In many cultures, women are frequently exposed to additional discrimination as they are traditionally perceived as a vulnerable population. Since CC is a gender-specific condition, women who typically hold household responsibilities and major support to their families tend to refrain from receiving a seemingly ‘invasive’ procedure like the Pap test unless symptoms emerge (Azaiza and Cohen 2008; Kelly et al., 2015). Additionally, socioeconomic status (SES) generates variations in CC screening, irrespective of country’s level of development (Massat et al., 2015; Todorova et al., 2009; Chiou et al., 2014; Mukem et al., 2015; Sabates and Feinstein 2006). Disparities caused by different ethnicity, education, urbanization, and income are still present worldwide. Impact of education and geographical area on CC screening uptake has been proven significant (Walsh et al., 2011; Todorova et al., 2009; Chiou et al., 2014; Sabates and Feinstein 2006; Wang and Lin 1996). Occupation is a common indicator for SES and was considered a predictor for screening uptake since women who are unemployed were seen less likely to receive a Pap test (Wang and Lin 1996). Nevertheless, to our knowledge, current evidence on the differences in CC screening inherent to occupational classes in females is still scarce. More importantly, even less research has examined the ensuing disease burden and economic costs as a result of these disparities.

Here we aim to first measure the extent of occupational inequalities in CC screening among Taiwanese women. Our second objective is to assess the health and economic benefits forgone as a result of these avoidable differences under the national screening policy. 

## Material and Methods

*Data source*

We identified a nationally-representative sample of females aged 30 years or above who are eligible for Pap smear testing in Taiwan from 1 January to 31 December 2013. Source of our data was an administrative database containing a nationally representative sample of beneficiaries from the National Health Insurance Research Database (NHIRD). Taiwan has a population approximately 23 million people, over 99% of which are enrolled in the universal National Health Insurance (NHI) program. This database was designed for the country’s single-payer system to manage all medical claims and was released for research purposes under restricted conditions. Approval from the institutional ethics review board was obtained before study commenced (CMUH105-REC1-077).

*Measurements*

For the purpose of this study, SES of the female subjects was defined using the occupation classification as specified in their NHI registration, which was listed by the nature of their employers or insurers. Consequently, we identified females aged 30 years or above who were employed at the time of observation. Two particular occupation groups were focused in this study: classes O1 and O5. The former (O1; the “general” group) consists of employed individuals, public and private employees, employers, or self-employed with specialized skills which comprised the majority of the productive workforce in the country. The latter (O5; the “low-income” group) consists of members from low-income households whose monthly income, despite their employed status, did not reach the lowest living standard in the country and required social welfare support from local government authorities as per Taiwan’s Public Assistance Act. In 2013, in order to be qualified with a ‘low income’ status, one must earn an average monthly income of between New Taiwan Dollars (NT) $6,829 (US$299.40) to NT$10,244 (US$344.10) per person and not over, which is below the national lowest living standard (Ministry of Health and Welfare 2018). This income is calculated by dividing the total monthly household income by the number of household members. Other occupation classes were not considered in this study since their SES could not be effectively represented given the variability and potential misclassification error intrinsic to the nature of the data source. Examples of these individuals include trade union workers or workers on contracts. 

Since subsidized screening is provided every three years, women who have received Pap smear within the 3-year timeframe up to 2013 were considered ‘screened’. Screening rate was calculated by dividing the number of screened by the total number of females eligible for screening. For 1-year screening rate, only those screened in the 1-year timeframe up to 2013 were considered.

Females who were ever diagnosed with the International Classification of Diseases, Ninth Revision, Clinical Modification (ICD-9-CM) code of 180 and were still active NHI enrollees in 2013 were defined as cervical cancer (CC) cases. Prevalence was estimated by dividing total number of identified CC cases by the total number of females eligible for screening. To estimate how many number of CC cases were actually identified among those screened, we used detection rates derived from dividing the number of CC cases by the total number of screened individuals. Detection rate is considered as the ability to detect (positive test) patients with the condition among all those that tested.

Deaths attributable to CC were defined using several assumptions: first, death during admission as indicated in the medical records; second, death as identified in catastrophic illness health records, or; third, NHI disenrollment within 28 days after discharge from CC-attributable admission (i.e. against-advice discharge). Only deaths in 2013 were included. Death rate was calculated by dividing the number of CC-attributable deaths by the total number of females eligible for screening in the same year. 

Health care expenditure was examined as an aggregate of all health care costs for each individual following their initial CC diagnosis up until the end of observation (e.g. 31 December 2013). Because we believe a majority of spending may come from admission costs, we also specifically examined the sum of all incurred inpatient costs subsequent to diagnosis for CC cases up to the end of observation. Cost values are reported in US dollars (US$1 = NT$29.77 in 2013). 

*Statistical analysis*

We used Poisson regression models to test the impact of occupational differences on CC prevalence and death rates after accounting for screening history. Models were further adjusted for age of CC diagnosis. The effect on health outcome and 95% confidence intervals (CIs) were reported. All statistical analyses were done using SAS 9.4 software package. 

*Estimation of economic impact on screening inequalities*

We first quantified health disparities in terms of differences in the 3-year and 1-year screening rates, CC prevalence rate, CC-attributable death rate, total health care costs per CC patient and hospital admission costs per CC patient. This was done by simply subtracting the estimates we have for O5 by that of O1 (i.e. O5-O1). We were then able to calculate the number of females affected by these disparities in the entire population using the total number of females found in our sample and multiplied that by 23 (i.e., 1 million random sample in our study is roughly 1/23 the size of the country’s total population). 

As a reference to the country’s economic status, gross domestic product (GDP) in 2013 was used and obtained in US dollars from the Directorate General of Budget, Accounting and Statistics, Executive Yuan. Health care expenditure in 2013 (total and for hospital admissions) was collected from the Ministry of Health and Welfare statistics report and expressed as a percentage of GDP. 

To estimate the forgone economic benefits associated with inequalities in CC screening, two different methods were used. We first considered health as a ‘consumption’ good (Mackenbach et al., 2011). This approach regards health as an individual investment good or utility where being in good health makes one feel satisfied or happy. Here we used ill health due to CC, CC-attributable death and potential health gains from screening to measure the value of health as a consumption good. Secondly, we considered health as a ‘capital’ good (Mackenbach et al., 2011). This method takes on a broader perspective where health in individuals is viewed as a common societal good such that people are able to work and generate income while being healthy. Here we used productivity and social benefits to measure the value of health as a capital good. Productivity was estimated using wages and income, i.e., the product of total >30y females in labor force and their average annual income in 2013. Social benefits were measured as a sum of unemployment compensation, as well as early reemployment allowance and parental leave allowance from the Bureau of Labor Insurance in the same year. 

*Sensitivity analyses*

To test the robustness of our results, we performed four scenario analyses with different alternative situations. First, we assumed that screening rates for both occupation groups are identical such that the screening rate in the lower group is levelled up to that of the higher screening group (O5=O1). Second, the screening rate is ubiquitously increased by 30% in both occupation groups (O1 and O5 groups both +30%). Third, the occupational difference in screening rate becomes 30% smaller than originally observed. Lastly, additional to conditions applied in Scenario 3, we further adjusted the detection rate in O5 to be no different than that of O1 (i.e., lowered detection rate may imply that individuals are more likely to receive screening as a preventive measure before becoming sick, and less likely to receive screening as a reaction to disease or disease-like symptoms, thus less cases detected among those screened).

## Results

*Pap testing rate, cervical cancer, and health care costs*

A total of 301,057 enrolled females aged 30 years and older eligible for screening were identified from our one-million population-based random sample in 2013. After stratification, 55.2% (n=166,101) and 0.34% (n=1,017) of subjects belonged to the general occupation (O1) and low income (O5) groups, respectively (Table 1). 

Overall, 3-year and 1-year screening rates among all subjects were 0.601 (181,063/301,057) and 0.372 (111,935/301,057), respectively. CC prevalence rate was 0.00425 (1,280/301,057) and CC-attributable death rate was 0.00008 (25/301,057) (column 1). When compared O5 with O1, we found that screening rates were consistently lower in the former: 0.568 (vs. 0.591) for 3-year screening, and 0.349 (vs. 0.359) for 1-year screening. Although negligible, the prevalence rate of cancer was nevertheless slightly higher in O5 (0.00393) relative to O1 (0.00345). Median total health care cost per CC patient following diagnosis was approximately US$6,673 for O5 and US$6,026 for O1 females (a difference of US$647). Uniquely, death rate was 0.00007 in O1 whereas no CC-death was detected in the O5 group. This could be attributable to the nation’s low CC mortality rate and the small group size identified for O5.

As a result of the empirically observed differences (column 4), we were able to infer the magnitude of these inequalities on the entire population as if we had the entire 23-million population (column 5): an occupational gap of 524 females screened within a 3-year period, 221 screened in the previous year, and 11 CC cases diagnosed. Moreover, we found that detection rate for CC (number of CC cases found among screened females in the last 3 years) was slightly higher in low-income females: 5.8/1,000 (573/98,119) and 6.9/1,000 (4/578) for O1 and O5, respectively. 

*Occupational differences in the impact of screening status on health*

Results from our Poisson regression model with 3-year and 1-year screening showed an increased risk of being diagnosed with CC (effect of 1.41, 95% CI: 1.13-1.75 and effect of 1.39, 95% CI: 1.12-1.73, respectively) in O5 females compared with O1 counterparts, after adjusting for age and the impact of screening with consideration of other occupational groups (Table 2). Interestingly, the relative risk of developing CC was also higher in females who previously received screening when compared with those who had not screened in the observed period (Model A for 3-year 2.15, 95% CI: 1.89-2.46; Model B for 1-year: 1.89, 95% CI: 1.69-2.11). Unfortunately, we could not provide a robust analysis examining the relative risk of CC-death attributable to occupational differences due to the small number of deaths detected. 

*Economic implications as a result of inequalities in Pap testing*

We estimated the impact of CC-screening inequalities on health care expenditure to reach US$59,568 in total health care costs in 2013, 90.4% (US$53,862) of which was specific to hospital admissions (Table 3). According to the official labor statistics, the average annual income for a female in 2013 was almost US$16,395 (Ministry of Labor 2018).

When we considered health as a ‘consumption’ good, the impact on total GDP from health losses through CC totaled to around 0.000015% (US$78,095). For ill health due to CC, our impact of inequalities here was estimated to be 11 persons which is equivalent to a monetary value of US$16,170. We derived this assuming US$100,000 per life-year according to Nordhaus (2002). Yang et al., (2004) estimated that there is a 14.3 years of life lost (YLL) per 1,000 people due to CC in women aged between 25 and 64 from East Asian countries. For the value of disparity in 3-year screening, we calculated it using the impact of inequalities (difference in no. screened = 524) from Table 1 and estimated it to be US$61,925. This was based on a CC prevalence rate of 10.1% in the country (Chiang et al., 2010), 81.9% sensitivity of Pap smear test for successfully detecting CC (Chao et al., 2008), value of 1 life-year saved assumed at US$100,000 per life-year (Nordhaus, 2002) and a total of 14.3 years of YLL per 1,000 due to CC (Yang et el. 2004). That is, if we had 524 more O5 females screened, 10.1% of them may have CC and the screening test would be able to detect 81.9% of them. For these detected individuals, their health could be potentially ‘saved’ through early detection, assuming that the disease would generate 14.3 years of YLL/1000 on average and each life year would be valued at US$100,000. 

As we considered health as a ‘capital’ good, we found that CC screening inequality-related losses in productivity or wages and income to be approximately US$185,391 or 0.000036% loss in GDP. This should be interpreted as an economic loss from those women who are sick with CC and was estimated using the product of inequalities in CC cases (n=11) and the average annual wage for females 30 years and above in 2013 (Ministry of Labor 2018). Similarly, the costs of social benefits due to impact of screening inequalities were about US$5,476, based on the year-, sex- and age-specific benefit payments provided by the Bureau of Labor Insurance in 2013. A total of US$190,868 (0.000037% of GDP) becomes the value of loss in capital as a consequence of CC inequalities.

*Scenario analyses*

We first presented our findings from the base case in terms of the inequalities resulted from occupational differences (Table 4). For instance, from table 1, we were able to estimate a gap of 524 females that resulted from the difference in 3-year screening rate and a gap of US$59,568 of total health care expenditure between O1 and O5. Also, from table 3, a difference of US$185,391 in productivity was generated from the product of number of CC females affected in the population (i.e., 11 from Table 1) and the annual average wage earned by a female in 2013. Similarly, a difference of US$5,476 in social benefits was estimated as the product of number of CC females affected in the population and the annual amount of social benefits provided for each female case, respectively. 

Sensitivity analyses with different screening scenarios revealed that when the gap in screening is eliminated between occupational groups (scenario 1), a total of US$24,176 of health care spending would be saved in a year, as well as US$1,755 in social (unemployment) benefits (Table 4). More importantly, we estimated that a considerable increase in productivity of up to US$59,397 in value would be observed among the female (>30y) population. In the second scenario, we assumed that screening rates of the two occupation groups were both increased by 30%, and this resulted in an augmented gap in the number of screened females between groups (681 vs. 524 in base case) (i.e., more O1 individuals ended up screening). Consequently, this led to a slightly higher health spending, greater social benefit payments, and also a decreased productivity (scenario 2). In the third scenario, we considered the size of screening gap to be reduced by 30% in comparison with the base case. This resulted with a preferable drop in the health inequality seen in absolute number of screened females (366 vs. 524 in base case). Health care costs (US$7,253) and social benefit payments (US$526) declined, and productivity was improved by US$17,819 (scenario 3). Finally, in the fourth scenario, we assumed scenario 3 with an additional change in cancer detection rate for which low-income females now had the same detection rate as the general occupation group. This would imply that for every 1,000 females screened, less positive cases would be diagnosed in low-income women. As a result, we found that the decrease in health inequality in the number of screened females was identical to scenario 3. Health care costs (US$6,120) and social benefit payments (US$444) saved, however, were not as high as in the previous scenario, and the increase in productivity (US$15,037) was also not as profound (scenario 4).

## Discussion

Our results suggest that occupational differences in CC screening can generate substantial forgone health and economic benefits. Under the current national screening policy, we detected a 3-year screening rate of 60.1% overall, with lower rate observed in low-income occupation females when compared to the general occupation group. CC prevalence rate and per patient health care spending were also higher in low-income women. Relative to the general group, low-income females were more likely to be diagnosed with CC, even after adjustment for age and screening history. 

We noted that the impact of observed differences in screening translated to US$59,568 (~3.6 times the female average annual income) of health care spending in one year, 90.4% of which was specific to hospital admissions. When we viewed health as a consumption good and capital good, the impact of screening disparity on health losses through CC would be equivalent to a total of US$78,095 and US$190,868, respectively. From our sensitivity analyses, we observed that the best scenario (i.e., decreased health care costs and social benefit payments together with increased productivity) was when we levelled up the screening rate of the low-income group so that there is no screening inequality between the two groups. Conversely, the gap widens and loss in productivity exacerbates if we chose to improve screening uptake in both occupation groups. Nevertheless, it is important to note that all provided estimates are annual values for the entire population, so they will continue to accumulate if the health inequalities persist. 

Three-year screening rate obtained from this study was slightly higher than what was found in earlier studies (~ 51.0%) which suggests a rise in screening uptake among Taiwanese women (Chen et al., 2009). Interestingly, we were also able to find out that there are 7.1 CC cases in 1,000 screened (i.e., 1,280 detected cases out of 181,063 screened). Although we were not able to see any notable differences in CC mortality between the two occupation groups, we were able to reveal that there exists a screening disparity which can be interpreted as a difference in participation or health-care accessibility to Pap test in part influenced by the nature of occupation in females. This was supported by previous studies which attribute differential access to cancer screening to individual-level SES (Todorova et al., 2009; Coughlin et al., 2006; Damiani et al., 2012) and partially informed choices (Goldie and Daniels 2011). A French study indicated that women in lower occupational class were more likely to have a delay in CC screening of over 3 years (Rigal et al., 2011). For Asian women, culturally sensitive care may also hinder access. 

Unfortunately, not many studies have examined the economic consequences of screening inequality. Although we found only minor impact on the country’s GDP from health losses through CC (health as consumption good), it is equivalent to many years of income generated by a woman, and a majority share of it (US$61,925 out of US$78,095 per year) could be potentially avoided if we diminished the gap in screening uptake. When we further examined health as human capital, an even greater loss was observed as much of it was attributed to productivity loss owing to the inequalities. A Polish study estimated a substantial level of CC-attributable annual productivity loss equivalent to approximately 702,964 working days lost or 111.4 million Euros due to women’s disability and mortality (Dubas-Jakóbczyk et al., 2016). 

Although previous studies had illustrated disparities in CC screening and outcomes, this study, however, extends past research by highlighting the economic implications associated with inequalities in CC screening participation. More importantly, the use of occupation classification as the social indicator in specifically women is rarely observed. Furthermore, a nationally-representative sample was used herein to derive the measurements, with multiple analytical methods used, including scenario analysis to test the robustness of our results. Lastly, evidence from the current study can be readily presented to policymakers and the public for awareness to be raised.

**Table 1:** Pap Smear Testing Rates and Related Health Status for Taiwanese Females >=30y by Occupation in 2013

	Overall (1)	Occupation 1 (O1) (2)	Occupation 5 (O5) (3)	Impact of health inequalities (O5-O1) (4)	No. females affected in population (5)
	301,057 (100.00%)	166,101 (55.2%)	1,017 (0.34%)	--	
Absolute number of screened (last 3 years)	181,063	98,119	578	--	
3-yr screening rate	0.601	0.591	0.568	-0.022	524
Absolute number of screened (previous year)	111,935	59,549	355	--	
1-yr screening rate	0.372	0.359	0.349	-0.009	221
Number of CC cases	1280	573	4	--	
CC prevalence rate	0.00425	0.00345	0.00393	0.00048	11
Number of CC deaths	25	11	0	--	
CC death rate	0.00008	0.00007	0	-0.00007	NA
Mean age at CC-attributable death (y)	73	73	NA	NA	
Total health care costs per CC patient, US$ (median)	6,393	6,026	6,673	647	59,568
Hospital admission costs per CC patient, US$ (median)	1,692	1,581	2,167	585	53,862

CC, cervical cancer; US$, US Dollars

**Table 2:** Poisson Regression Estimation of the Occupational Differences in the Impact of Screening Status on Health Outcomes (2013 only)

	CC Case		CC Death	
Summary measure of effect	Effect	95% CI	Effect	95% CI
Model A*				
Screened (3-yr)				
No	1	Reference	1	Reference
Yes	2.15	(1.89-2.46)	5.48	(1.63-18.42)
Occupation				
Occupation 1	1	Reference	1	Reference
Occupation 5	1.41	(1.13-1.75)	NA	NA
Model B*				
Screened (1-yr)				
No	1	Reference	1	Reference
Yes	1.89	(1.69-2.11)	5.09	(2.03-12.80)
Occupation				
Occupation 1	1	Reference	1	Reference
Occupation 5	1.39	(1.12-1.73)	NA	NA

CC, cervical cancer; CI, confidence interval; NA, not available; *, Models adjusted for age with consideration of other occupational groups; *interpreted as the relative change in health outcome per unit increase in screening case number

**Table 3:** Forgone Health and Economic Benefits Associated with Inequalities in Cervical Cancer Screening Uptake, 2013

	Overall		Result of inequalities (from Table 1)	
	Estimate (million US$)	As % GDP	Estimate (US$)	As % GDP
GDP, 2013	511,614	100.00%	--	--
Health care expenditure, 2013				
Total	32,512	6.35%	59,568	1.2x10^-5^ %
Hospital admissions	5,174	1.01%	53,862	1.1x10-^5^ %
Health as ‘consumption’ good				
CC death	NA	NA	-	-
CC case	NA	NA	16,170	3.0x10^-6^ %
3-yr screened	NA	NA	61,925	1.2x10^-5^ %
Total	NA	NA	78,095	1.5x10^-5^ %
Health as ‘capital’ good				
Productivity (wages and income)^a^	82,680	16.20%	185,391	3.6x10^-5^ %
Social (unemployment) benefits^b^	245	0.05%	5,476	1.0x10^-6 ^%
Total	82,925	16.20%	190,868	3.7x10^-5^ %

US, United States; GDP, gross domestic product; CC, cervical cancer; NA, not available; ^a^, only productivity estimates for females 30 years and over were obtained for this analysis; ^b^, include unemployment benefits, as well as early reemployment allowance, parental leave allowance; US$1, NT$29.77 in 2013.

**Table 4.** Scenario Analysis with Alternative Screening Uptake Options

	Scenarios				
Yearly estimates	Base case (as observed in tables 1 and 3)	Scenario 1: Screening rate is ‘levelled up’ to that of observed in O1 (i.e. no inequality in screening)	Scenario 2: Screening rate is ubiquitously ‘levelled up’ by 30% in both O1 and O5	Scenario 3: Occupational differences in screening are 30% smaller than observed	Scenario 4: Scenario 3 + detection rate in O5 same as that of O1
3-yr screened (no.)	524	0	681	366	366
Total health care expenditure (US$)	59,568	-24,176	7,253	-7,253	-6,120
Productivity (US$)	185,391	59,397	-17,819	17,819	15,037
Social benefits (US$)	5,476	-1,755	526	-526	-444

However, our estimates should be interpreted with caution as they are not without limitations. The economic value for per life saved used was based on the work from Nordhaus with a willingness-to-pay study performed in the US, which could differ in the setting under study. Moreover, we compared lowest-income working females with a sample of general occupation females, rather than those from the highest income group. This suggests that the disparity between the most affluent and the poorest could potentially be larger. Female productivity was calculated based on country statistics from 2013 after which both the female monthly wage and its share of labor force have been gradually growing. Since we only acquired data from a single year, the very low CC-attributable mortality in the country made it difficult to illustrate a gap in CC deaths. Histology of the cancer (squamous cell carcinoma or adenocarcinoma) may also affect the mortality rate which we could not depict from our data.

Forgone health and economic benefits associated with inequalities in CC screening uptake can be considerable in productive women. As the female role becomes more prominent in the modern workforce, it becomes imperative to look closely into the impact of their illness attributable to avoidable disparities from the societal and economical perspective. Collaborative efforts from policy-makers and providers should be actively promoted to reduce occupational differences in CC screening in practical settings. Access to cancer screening and other secondary prevention interventions should be truly based on fully informed choices irrespective of socioeconomic classes. 

*Summary*

1. Women being still bound by household obligations and the culturally-sensitive nature of Pap tests are two possible factors that hinder access to cervical cancer screening and effective cancer prevention in this vulnerable population. 

2. This study extends past research by highlighting the economic implications associated with inequalities in cervical cancer screening uptake. 

3. Impact of screening inequalities from different occupation groups on health care expenditure reached US$59,568 in total health care costs in 2013, 90.4% (US$53,862) of which was specific to hospital admissions.

4. Majority of health losses as a consumption good from cervical cancer was attributable to inequalities in screening participation, and inequality-related losses in human capital was estimated to be approximately US$190,868 per year.

5. Among different scenarios tests, the best resolution is to reduce the gap in screening between occupation groups as it would generate the most savings in health care cost and social benefits, as well as the highest increase in labor productivity. 
